# Determination of Sunset Yellow and Tartrazine in Food Samples by Combining Ionic Liquid-Based Aqueous Two-Phase System with High Performance Liquid Chromatography

**DOI:** 10.1155/2014/964273

**Published:** 2014-11-05

**Authors:** Ou Sha, Xiashi Zhu, Yanli Feng, Weixing Ma

**Affiliations:** ^1^Analysis and Test Centre of Jiangsu Marine Resources Development Research Institute, Lianyungang 222001, China; ^2^College of Chemistry & Chemical Engineering, Yangzhou University, Yangzhou 225002, China; ^3^School of Chemistry and Chemical Engineering, Huaihai Institute of Technology, Lianyungang 222005, China

## Abstract

We proposed a simple and effective method, by coupling ionic liquid-based aqueous two-phase systems (IL-ATPSs) with high performance liquid chromatography (HPLC), for the analysis of determining tartrazine and sunset yellow in food samples. Under the optimized conditions, IL-ATPSs generated an extraction efficiency of 99% for both analytes, which could then be directly analyzed by HPLC without further treatment. Calibration plots were linear in the range of 0.01–50.0 *μ*g/mL for both Ta and SY. The limits of detection were 5.2 ng/mL for Ta and 6.9 ng/mL for SY. This method proves successful for the separation/analysis of tartrazine and sunset yellow in soft drink sample, candy sample, and instant powder drink and leads to consistent results as obtained from the Chinese national standard method.

## 1. Introduction

Tartrazine (Ta) and sunset yellow (SY) are synthetic food colors used most extensively as food additives, to improve the appearance, color, and texture of foods [1]. When added in excess, however, these synthetic food colors can be pathogenic [2]. To ensure food safety, the Chinese government has imposed rigorous standards on the permitted levels for various synthetic food colors [3]. For the same reason, it is important to develop effective methods for analyzing synthetic colors in food.

Various techniques have been applied for analyzing synthetic colors in food samples, including spectrophotometry [4], capillary electrophoresis [5], differential pulse polarography [6], liquid chromatography [7], and others [8], which all require an optimal extraction method to concentrate an analyte from a small amount of food sample containing miscellaneous components [9, 10]. Solid phase extraction (SPE) and liquid-liquid extraction (LLE) have been reported for separating synthetic food colors from different matrices [11, 12]. For SPE, analytes are separated following complicated and time-consuming absorption and desorption steps involving the use of toxic and volatile reagents, such as methanol, acetic acid, and ammonia, that are used in the current Chinese national standard method [13]. In contrast, an aqueous two-phase system (ATPS) has attracted increasing attention in that it minimizes the potential interferences from other components and enables the simultaneous extraction and concentration of analytes [14]. By far, most reported ATPSs are generated by mixing two solutions of polymers, such as dextran T500 and polyethylene glycol (PEG) system [15], or by adding a high-concentration salt solution to an aqueous polymer solution [16]. The partition behavior of analytes between the polymer-rich phase and the aqueous phase can be controlled and optimized with a judicious choice of phase system. However, most of polymer-rich phase is highly viscous and opaque, rendering the follow-up analysis difficult. To address these issues, the development of a simple and environmentally friendly method with minimal use of volatile and toxic solvents used is of great importance.

Ionic liquid (IL) is a green solvent and a potential replacement for traditional volatile solvents [17]. In 2003, Gutowski and coworkers first generated several ATPSs from different IL and salts and determined the corresponding phase diagrams [18]. The identity of IL is important for the separation/analysis of a specific analyte in IL-based ATPS [19]. An optimal IL-based APTS should possess the following features: minimal emulsion formation, low viscosity, rapid phase separation, high extraction efficiency and low cost, and so forth [20]. In this study, we aim to develop a simple and “green” IL-based ATPS extraction method with high efficiency and couple it with HPLC for the analysis of Ta and SY from food samples. For this purpose, we selected 1-butyl-3-methylimidazolium bromide ([C_4_MIM]Br), which is not easy to emulsify and presents a lower viscidity compared with polymer-salt ATPS. Besides, [C_4_MIM]Br was diffluent in water and methanol (the HPLC mobile phase). Analytes (Ta + SY) could be extracted into IL phase and analyzed by HPLC with no further sample treatment. We showed the success of this method in the separation/analysis of Ta and SY from soft drink sample, candy sample, and instant powder drink.

## 2. Experimental

### 2.1. Reagents and Apparatus

All reagents were of analytical grade or higher in purity and purchased from Sinopharm Chemical Reagent Co., Ltd., Shanghai, China, unless otherwise specified. HPLC grade methanol was purchased from Merck (Germany). The standard stock solutions of the colorants, tartrazine (Ta; C.I. Food Yellow 4; 0.5 mg/mL), and sunset yellow (SY; C.I. Food Yellow 3; 0.5 mg/mL) were obtained from the National Research Center for Certified Reference Materials (Beijing, China) and were both prepared in distilled water to a final concentration of 100 *μ*g/mL. All glass containers were stored in 10% (v/v) nitric acid overnight and rinsed with distilled water before use. ILs (1-alkyl-3-methylimidazolium bromide, [C_*n*_MIM]Br, *n* = 4, 6,8) were synthesized as described previously [21].

All spectral measurements were carried out using a model UV-2501 spectrophotometer (Shimadzu, Japan). Phase separation of the sample solution was achieved with a centrifuge (Model TDL80-2B, Shanghai Anting Science Instrument Factory, Shanghai, China). Chromatographic analyses were performed on an Agilent 1260 HPLC system which consisted of a 1260 infinity quaternary pump with degasser gradient pump, a 1260 infinity variable wavelength detector, a 1260 infinity manual injector, and an open LAB CDS chemstation workstation (Agilent, USA).

### 2.2. Preparation of Phase Diagram

Phase diagrams were determined using the cloud-point method [22]. A certain of [C_*n*_MIM]Br was put into a 10.0 mL centrifugal tube. K_2_HPO_4_ solution or the other tested salt solution was added dropwise to the test tube until a turbidity formed, indicating the formation of a two-phase system. Thereafter, water was added dropwise to the test tube to obtain a clear one-phase system. More salt solution was added again to afford a two-phase system. The composition of this mixture was noted and so on. The bimodal curve was applied to characterize the phase diagram [23]. In the region above the bimodal curve, the system is divided into two phases; in the region below the bimodal curve, the system is of a homogeneous phase.

### 2.3. IL-Based ATPS Extraction

0.3 mL of [C_4_MIM]Br and 1.0 mL of the mixed standard solution (100 *μ*g/mL SY + 100 *μ*g/mL Ta) or 1.0–3.0 mL of the sample solution were placed in a 10.0 mL centrifugal tube. The mixture was diluted to 4.0 mL with distilled water and then 3.0 g K_2_HPO_4_ was added. The phase separation quickly occurred in the tube after gentle blending. The tube was centrifuged for 2 min at 3500 rpm to ensure a complete phase separation. 20.0 *μ*L of [C_4_MIM]Br phase was withdrawn using a microsyringe and injected into HPLC for quantification.

### 2.4. Chromatographic Conditions

Chromatographic separation was achieved on a Zorbax ODS column (4.6 mm × 150 mm × 5*μ*m) associated with a guard column packed with the same bonded phase. The composition of the mobile phase at time zero (the time of injection) was 85% ammonium acetate (20 mM, pH 4.5) and 15% methanol. Over the next 5 min, the percentage of methanol was gradually increased to 40%. Thereafter, the mobile phase was changed to ammonium acetate-methanol (2 : 98; v : v) within 5 min. Finally, the chromatographic system was equilibrated during 5 min before the next injection. A flow rate of 0.8 mL/min was used throughout the 15 min run. Chromatography was performed at 30°C. Dual UV wavelength mode was used, with Ta monitored at 420 nm and SY at 480 nm. The mobile phase was filtered through 0.45 *μ*m micropore filter membrane prior to use.

### 2.5. The Extraction Parameter

The distribution behaviors of Ta and SY between IL phase and salt phase were characterized by the extraction efficiency (*E*), partition coefficient (*K*), and phase ratio (*R*).

The parameters *E*, *K*, and *R* were defined as follows:

(1) $$\begin{array}{c} E=\frac{C_{\text{IL}}V_{\text{IL}}}{C_{\text{aq}}V_{\text{aq}}+C_{\text{IL}}V_{\text{IL}}}\times 100\%,\\ K=\frac{C_{\text{IL}}}{C_{\text{aq}}},\\ R=\frac{V_{\text{IL}}}{V_{\text{aq}}}, \end{array}$$

where *C*_IL_ and *C*_aq_ are the concentration of Ta or SY in IL phase and in salt phase, respectively, and *V*_IL_ and *V*_aq_ are the volume of IL phase and K_2_HPO_4_ phase, respectively.

The spectra and absorbance of Ta or SY in [C_4_MIM]Br/K_2_HPO_4_ system were determined as an example to study the distribution behavior and extraction efficiency. During each experiment, the IL phase containing the analyte was collected to measure the UV-visible spectra on the UV-2501 UV-vis spectrophotometer. The absorbance of Ta and SY was measured at 420 nm and 480 nm, respectively. The blank containing the same phase composition but without analyte was used as reference solution.

### 2.6. Sample Analysis

All samples, including soft drink, candy, and instant powdered drink, were obtained from a local market. Appropriate amounts (0.1–5 g) of the samples were dissolved in 15 mL of water. The carbonated drinks were degassed by ultrasonication for 5 minutes to remove the carbon dioxide. A warming process (50°C, 30 min) was used for the complete dissolution of the sugar-based candy. These solutions were centrifuged and the upper solutions were filtered through 0.45 *μ*m micropore filter membrane. The filtrate was transferred to volumetric flask of 25.0 mL, volume adjusted to 25.0 mL using distilled water, and ready for IL-ATPS extraction.

The Chinese national standard method (GB/T 5009.35-2003) was also used for separation/analysis of Ta and SY in different samples. Briefly, the sample solution was adjusted to pH 6.0 with 200 g/L citric acid solution and heated up to 60°C. Then this solution was stirred with polyamide powder for 5 min and filtered through Buchner filter using double-decked filter papers. After filtering, the polyamide powder was washed with deionized water for 3–5 times, followed with the mixture of methanol-formic acid for 3–5 times. The colorants absorbed by polyamide powder were eluted with 15 mL of eluting solution (30% ammonia solution mixed with ethanol in the volume atio of 3 : 7), adjusted to pH 7.0 by acetic acid, and evaporated to near dryness, following which distilled water was added to the volume of 5.0 mL for HPLC analysis.

## 3. Results and Discussion

### 3.1. Phase Diagrams

**Figure 1:**
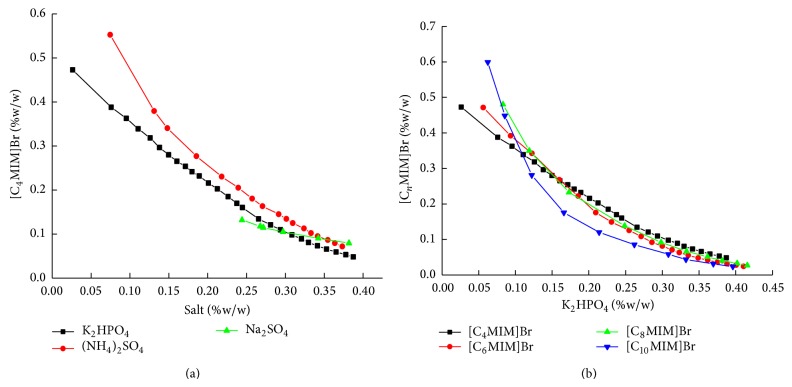
Phase diagrams of different salts (a) and [C_*n*_MIM]Br (b) for the ILs/salt/water systems at 25°C.

In this paper the bimodal curve for the aqueous two phase systems of [C_4_MIM]Br with a series of salts at 25°C was investigated. Two replicate measurements were performed for each point. Results showed that IL-salt ATPS could be formed by adding appropriate amount of different salts, such as (NH_4_)_2_SO_4_, Na_2_SO_4_, and K_2_HPO_4_. Other salts, such as K_3_PO_4_, KH_2_PO_4_, NaCl, and NaH_2_PO_4_, cannot separate [C_4_MIM]Br solution into two phases. As shown in Figure 1(a), the phase separation capability of salts is K_2_HPO_4_ > (NH_4_)_2_SO_4_ > Na_2_SO_4_, consistent with their solubility in water at 25°C, suggesting a correlation between the phase separation capability of salts and their solubility in water [24]. Consequently, K_2_HPO_4_ was chosen in the following studies.

Methylimidazolium bromides with four different carbon-chain length, namely, [C_4_MIM]Br, [C_6_MIM]Br, [C_8_MIM]Br, and [C_10_MIM]Br, were selected to investigate the effect of carbon-chain length on phase diagrams of IL-salt ATPS. The bimodal curves determined at 25°C for the IL/K_2_HPO_4_ system were shown in Figure 1(b). When the weight percentage of K_2_HPO_4_ was more than 0.15%, the phase separation capability of IL followed the order of [C_4_MIM]Br ≈ [C_6_MIM]Br ≈ [C_8_MIM]Br < [C_10_MIM]Br. In view of the lower synthetic cost and lower viscosity of [C_4_MIM]Br than that of [C_10_MIM]Br, [C_4_MIM]Br was chosen for further study.

### 3.2. Optimization of Extraction System

The extraction efficiency mainly depends on the structure of analyte and its affinity towards the extractant (i.e., partitioning coefficient), phase ratio, and the number of extractions in the liquid-liquid extraction system. It was also often used to estimate the migration ability of analyte between the two phases and the separation performance of extractant. Higher separation efficiency can be achieved with a greater partitioning coefficient *K* and a lower phase ratio *R* [25].

In this paper the effects of ionic liquid, salt, and temperature on extraction efficiency, partition coefficient, and phase ratio were assessed to obtain the optimal parameter.

#### 3.2.1. The pH Effect

**Figure 2:**
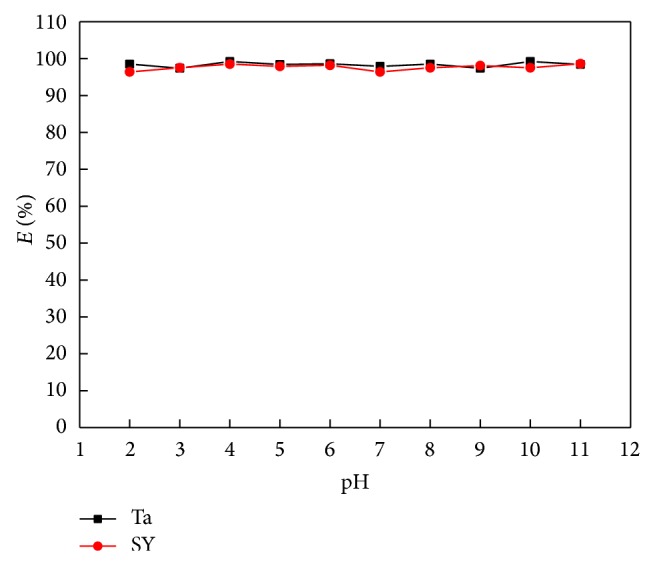
The effect of pH on the extraction efficiency (*m*_SY_ = 100 *μ*g, *m*_Ta_ = 100 *μ*g, *V*_IL_ = 0.30 mL).

The effect of pH on IL-ATPS extraction ability of [C_4_MIM]Br for Ta and SY was studied in the pH range of 2.0–11.0 by the addition of Britton-Robinson buffer solution in the presence of 0.3 mL [C_4_MIM]Br and 3.0 g K_2_HPO_4_ (Figure 2). It was foundthat the extraction efficiency remained relatively constant over the pH range and the pH has little effect on the extraction of Ta and SY.

#### 3.2.2. Effect of K_2_HPO_4_

**Figure 3:**
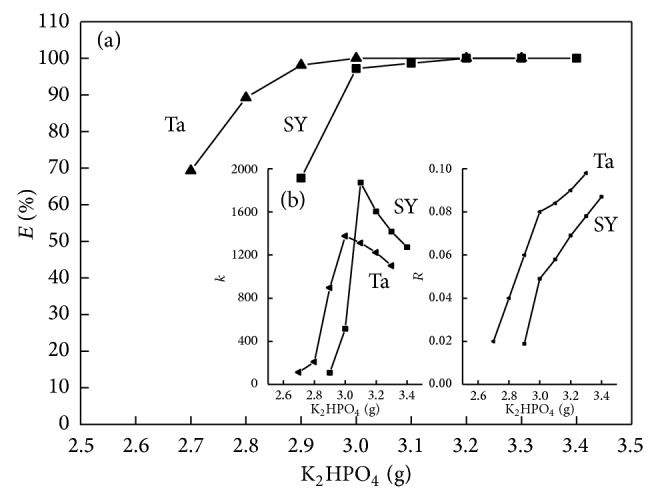
Effects of K_2_HPO_4_ amount on extraction efficiency, partition coefficient, and phase ratio (*m*_SY_ = 100 *μ*g, *m*_Ta_ = 100 *μ*g, *V*_IL_ = 0.30 mL).

With a fixed volume of [C_4_MIM]Br at 0.3 mL, [C_4_MIM]Br-K_2_HPO_4_ ATPS was formed when the amount of K_2_HPO_4_ was over 2.7 g in weight for Ta and 2.9 g in weight for SY, respectively. The influence of K_2_HPO_4_ concentration on the extraction efficiency, partition coefficient, and phase ratio was studied with 0.3 mL [C_4_MIM]Br added and the results obtained were shown in Figure 3. It revealed that the extraction efficiency obtained was above 95% with K_2_HPO_4_ of 3.00–3.40 g (Figure 3(a)). The partition coefficient of Ta and SY was increased to a maximum value when the amount of K_2_HPO_4_ was set at 3.0 g and 3.1 g, respectively, then decreased with the increased amount of K_2_HPO_4_. The phase ratio of Ta and SY was increased with the amount of K_2_HPO_4_ (Figure 3(b)). In order to achieve quantitative extraction and higher separation efficiency, 3.0 g of K_2_HPO_4_ was used in all following experiments for the simultaneous extraction of SY and Ta.

#### 3.2.3. Effect of [C_4_MIM]Br Amount

**Figure 4:**
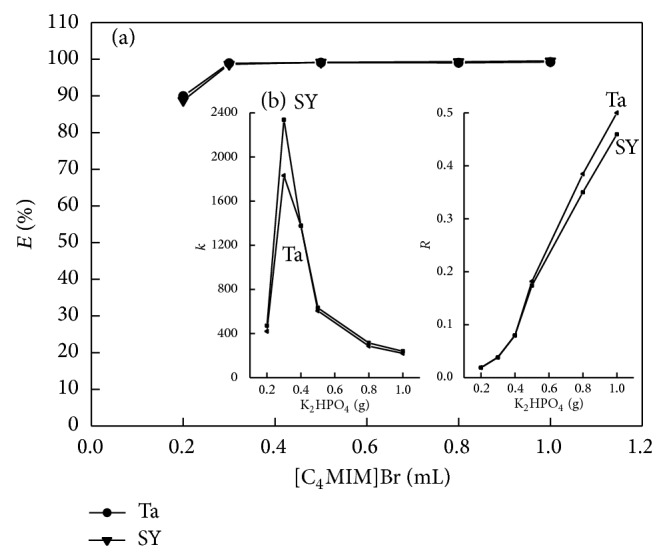
Effects of [C_*n*_MIM]Br amount on extraction efficiency, partition coefficient, and phase ratio (*m*_SY_ = 100 *μ*g, *m*_Ta_ = 100 *μ*g, *m*_K_2_HPO_4__ = 3.0 g).

The amount of [C_4_MIM]Br used in the preconcentration procedure is a critical factor for obtaining a high extraction performance. Therefore, the extraction system was carefully studied to determine the lowest IL-phase volume necessary for achieving the best extraction. In this system, the ATPS of [C_4_MIM]Br-K_2_HPO_4_ could not be achieved with 3.2 g K_2_HPO_4_ if the volume of [C_4_MIM]Br was less than 0.2 mL.So the effect of the volume of [C_4_MIM]Br on extraction efficiency was studied in the range of 0.2–1.0 mL. It was found that the extraction efficiency calculated was more than 95% in the concentration range of 0.3–1.0 mL (Figure 4(a)). The partition coefficient of Ta and SY got a maximum value when 0.3 mL [C_4_MIM]Br was added, and then decreased with amount of IL, whereas the phase ratio was increased with the increased amount of [C_4_MIM]Br (Figure 4(b)). Considering the extraction efficiency, enrichment factors, and the low consumption of [C_4_MIM]Br, 0.3 mL [C_4_MIM]Br IL was used to achieve a higher extraction efficiency in the subsequent experiments.

#### 3.2.4. Effect of Temperature

**Figure 5:**
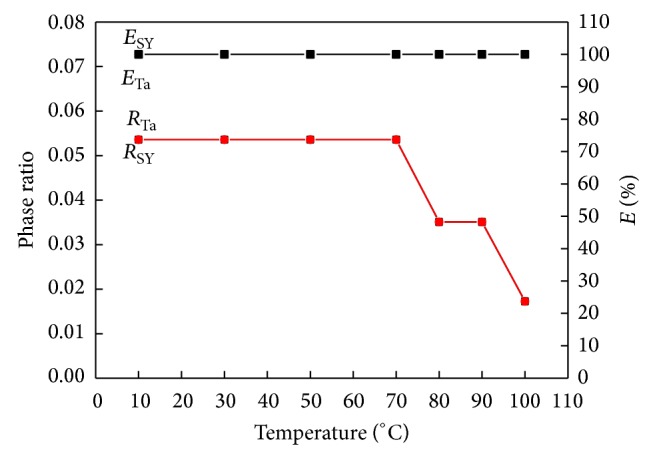
The effect of temperature on the extraction efficiency and phase ratio.

The effect of temperature on the extraction efficiency and phase ratio of Ta and SY by IL-salt APTS was also studied (Figure 5). The results revealed that the extraction efficiency of Ta and SY was near to 100% within the entire tested temperature range from 10°C to 70°C. The phase ratio was stable within this range and decreased when temperature was over 70°C. The curve of extraction efficiency and phase ratio of sunset yellow was superposed practically compared with that of tartrazine. The reason was that higher temperatures result in more [C_4_MIM]Br redissolved into K_2_HPO_4_-enriched bottom phase. Accordingly, [C_4_MIM]Br concentration in top phase decreased. But the decrease of [C_4_MIM]Br in top phase does not affect the extraction efficiencies of Ta and SY. This new extraction system can afford a wide temperature range for extraction of Ta and SY. In the following experiments, the temperature was set at room temperature of 25°C for the extraction of Ta and SY.

### 3.3. UV-Vis Spectrometric Studies of the Ta and SY in the [C_4_MIM]Br-Rich Top Phase

**Figure 6:**
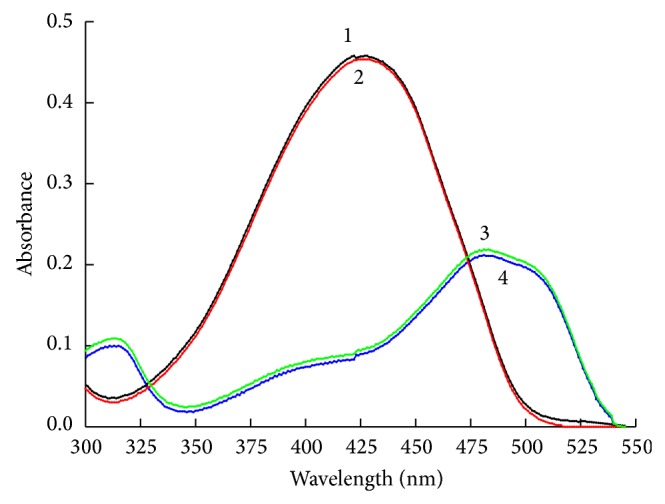
The UV-Vis spectra of colorants before and after extraction (*m*_SY_ = 100 *μ*g, *m*_Ta_ = 100 *μ*g, *V*_IL_ = 0.30 mL, *m*_K_2_HPO_4__ = 3.0 g).

The absorption spectra recorded for Ta (curve 1 and curve 2) and SY (curve 3 and curve 4) before and after [C_4_MIM]Br/K_2_HPO_4_ extraction, as scanned in the wavelength range of 350 nm–700 nm against the reagent blank, respectively, were shown in Figure 6. The spectra of these two colorants remained unaltered when they were extracted into the [C_4_MIM]Br-rich top phase and practically superimposed before and after [C_4_MIM]Br/K_2_HPO_4_ ATPS extraction. This observation clearly indicated that no direct chemical (bonding) interactions were involved between colorants and ionic liquid in the IL-ATPS.

### 3.4. Analytical Performance

Under the optimized conditions described above, analytical parameters of the proposed HPLC-UV-Vis method such as linearity, sensitivity, correlation coefficients, limits of detection, and precisions were evaluated. Calibration graphs were constructed by plotting the peak area versus the concentration of analytes. For the tested Ta and SY, linearity was observed in the concentration range of 0.01–50 *μ*g/mL. The other parameters are summarized in Table 1. The results demonstrate that the method is sensitive for the determination of Ta and SY in all studied samples. The intraday precision of the proposed method was tested with 6 repeated injections of solution containing Ta and SY standards at the concentration level of 0.5 *μ*g/mL. The relative standard deviations (RSD) were ≤3.2%. The obtained RSD values indicate a satisfactory precision of the proposed method. The interday precision, expressed as RSD of the slopes of the calibration graphs obtained in three different days, was ≤5.6%. Due to the fact that the RSD is higher than 5%, the calibration graphs should be registered the same day as the real sample is analyzed.

### 3.5. Sample Analysis

**Figure 7:**
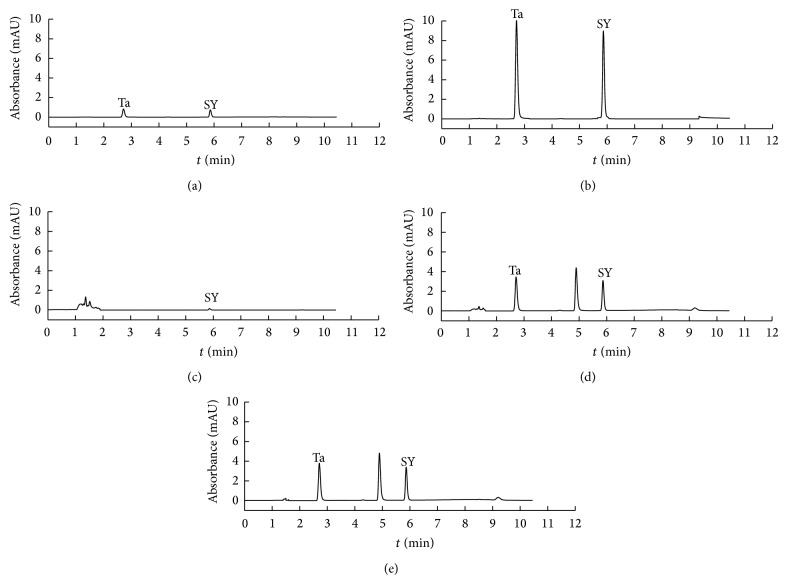
The chromatograms of standard solution and sample solution before and after extraction. (a) Standard solution of 0.25*μ*g/mL Ta and 0.25*μ*g/mL SY; (b) standard solution of 0.25*μ*g/mL Ta and 0.25*μ*g/mL SY after IL-salt ATPS extraction; (c) candy sample solution without pretreatment; (d) candy sample solution with polyamide adsorption method; (e) and candy sample solution with IL-salt ATPS (*V*_IL_ = 0.30 mL, *m*_K_2_HPO_4__ = 3.0 g).

Under the optimal conditions, the proposed [C_4_MIM]Br/K_2_HPO_4_ APTS-HPLC method has been adopted to determine Ta and SY in candy samples. Typical chromatograms of Ta and SY in the standard solution and candy sample were shown in Figure 7. Curves (a) and (b) were the chromatograms of the mixed standard solution (Ta + SY) before and after IL-salt ATPS extraction, respectively. Curve (c) was chromatogram of candy sample by direct sampling without treatment. Curves (d) and (e) were chromatograms of candy sample enriched by polyamide adsorption and IL-salt ATPS, respectively. Figure 7 showed that (1) the standard solution (curve (a)) and real sample (curve (c)) have been enriched (curves (b) and curve (d)) by IL-salt ATPS extraction; (2) the response signal of Ta and SY determined by IL-salt ATPS extraction-HPLC was consistent with GB/T 5009.35-2003 method (curves (d) and (e)).

The proposed procedure has been applied to the determination of Ta and SY in soft drink samples, candy samples, and instant powdered drink. The recoveries of Ta and SY in different food samples were in the range of 93.5–97.4% and 94.0–97.2%, respectively (Table 2). When compared with those achieved by the GB/T 5009.35-2003 method, as shown in Table 2, no significant difference was observed between the proposed method and GB/T 5009.35-2003 (*P* > 0.05).

## 4. Conclusion

A simple and rapid IL-based ATPS consisting of [C_4_MIM]Br and K_2_HPO_4_ coupled with HPLC-UV was developed for the sensitive and simultaneous determination of sunset yellow and tartrazine in soft drink samples and candy samples. In this research, direct injection of the [C_4_MIM]Br phase into HPLC system for the quantification of Ta and SY was proposed. This method proved to be efficient, simple and fast for separation/analysis of Ta and SY in soft drink samples, candy samples and instant powdered drink.

## Figures and Tables

**Table 1:** Analytical figures of merit for Ta and SY using HPLC-UV method.

Analyte	Slope of the calibration graph ±SD (*n* = 3)	Correlation coefficient *r*	Limit of detection (ng/mL)	Intraday precision RSD (%) (*n* = 6)^b^	Interday precision RSD (%) (*n* = 3)^a^
Ta	751.36 ± 2.14	0.9975	5.2	3.2	5.6
SY	838.51 ± 1.23	0.9967	6.9	2.4	4.8

^a^three independent calibration graphs obtained in three different days.

^b^concentration of analyte was 0.5 *μ*g mL.

**Table 2:** Determination of SY and Ta in food sample and recovery test (*n* = 3).

Sample	Ta^a^ (*μ*g/g)				SY^a^(*μ*g/g)			
	Added	Found	Recovery	GB-found^b^	Added	Found	Recovery	GB-found^b^
Soft drink 1	—	6.64 ± 0.14	—	6.51 ± 0.21	—	NT	—	ND
	5.0	11.46 ± 0.16	96.4%		5.0	4.86 ± 0.16	97.2%	
	10.0	16.38 ± 0.14	97.4%		10.0	9.45 ± 011	94.5%	

Soft drink 2	—	1.42 ± 0.24	—	1.38 ± 0.15	—	17.67 ± 0.32	—	17.09 ± 0.84
	2.00	3.32 ± 0.10	95.0%		10.00	27.18 ± 0.25	95.1%	
	5.00	6.21 ± 0.18	95.8%		20.00	36.59 ± 0.16	94.6%	

Soft drink 3	—	ND	—	ND	—	22.76 ± 0.23	—	21.86 ± 0.58
	5.00	4.76 ± 0.12	95.2%		15.00	36.98 ± 0.21	94.8%	
	10.00	9.41 ± 0.26	94.1%		20.00	41.88 ± 0.15	95.6%	

Candy 1	—	0.11 ± 0.05	—	1.11 ± 0.13	—	1.26 ± 0.15	—	1.20 ± 0.09
	2.00	2.07 ± 0.14	98.0%		2.00	3.19 ± 0.21	95.5%	
	4.00	3.85 ± 0.18	93.5%		4.00	5.04 ± 0.15	94.5%	

Candy 2	—	ND	—	ND	—	0.39 ± 0.02	—	0.38 ± 0.09
	0.50	0.47 ± 0.12	94.0%		0.50	0.86 ± 0.05	94.0%	
	1.00	0.94 ± 0.13	94.0%		1.00	1.34 ± 0.07	95.0%	

Papaya powder	—	ND	—	ND	—	1.50 ± 0.17	—	1.64 ± 0.09
	2.00	1.89 ± 0.10	94.5%		3.00	4.34 ± 0.13	94.7%	
	5.00	4.82 ± 0.18	96.4%		6.00	7.21 ± 0.12	95.2%	

${}^{\text{a}}\bar{x}\pm ts/\sqrt{n}$ at 95% confidence (*n* = 3).

^b^GB/T 5009.35-2003.

ND: not detected.

## References

[1] Kucharska M., Grabka J. (2010). A review of chromatographic methods for determination of synthetic food dyes. *Talanta*.

[2] Kamel M. M., El-lethey S. H. (2011). The potential health hazard of tartrazine and levels of hyperactivity, anxiety-like symptoms, depression and anti-social behaviour in rats. *Journal of American Science*.

[3] Khera K. S., Munro I. C. (1979). A review of the specifications and toxicity of synthetic food colors permitted in Canada. *CRC Critical Reviews in Toxicology*.

[4] Llamas N. E., Garrido M., Nezio M. S. D., Band B. S. F. (2009). Second order advantage in the determination of amaranth, sunset yellow FCF and tartrazine by UV-vis and multivariate curve resolution-alternating least squares. *Analytica Chimica Acta*.

[5] Dossi N., Toniolo R., Pizzariello A., Susmel S., Perennes F., Bontempelli G. (2007). A capillary electrophoresis microsystem for the rapid in-channel amperometric detection of synthetic dyes in food. *Journal of Electroanalytical Chemistry*.

[6] Chanlon S., Joly-Pottuz L., Chatelut M., Vittori O., Cretier J. L. (2005). Determination of Carmoisine, Allura red and Ponceau 4R in sweets and soft drinks by differential pulse polarography. *Journal of Food Composition and Analysis*.

[7] Alves S. P., Brum D. M., Branco de Andrade É. C., Pereira Netto A. D. (2008). Determination of synthetic dyes in selected foodstuffs by high performance liquid chromatography with UV-DAD detection. *Food Chemistry*.

[8] Silva M. L. S., Garcia M. B. Q., Lima J. L. F. C., Barrado E. (2007). Voltammetric determination of food colorants using a polyallylamine modified tubular electrode in a multicommutated flow system. *Talanta*.

[9] Pourreza N., Ghomi M. (2011). Simultaneous cloud point extraction and spectrophotometric determination of carmoisine and brilliant blue FCF in food samples. *Talanta*.

[10] El-Shahawi M. S., Hamza A., Al-Sibaai A. A., Bashammakh A. S., Al-Saidi H. M. (2013). A new method for analysis of sunset yellow in food samples based on cloud point extraction prior to spectrophotometric determination. *Journal of Industrial and Engineering Chemistry*.

[11] Zhang Z., Zhang Z., Fernández Y., Menéndez J. A., Niu H., Peng J., Zhang L., Guo S. (2010). Adsorption isotherms and kinetics of methylene blue on a low-cost adsorbent recovered from a spent catalyst of vinyl acetate synthesis. *Applied Surface Science*.

[12] Sharma Y. C. (2010). Optimization of parameters for adsorption of methylene blue on a low-cost activated carbon. *Journal of Chemical and Engineering Data*.

[14] Ruiz-Ruiz F., Benavides J., Aguilar O., Rito-Palomares M. (2012). Aqueous two-phase affinity partitioning systems: current applications and trends. *Journal of Chromatography A*.

[15] Hamamoto R., Kamihira M., Iijima S. (1996). Specific separation of animal cells using aqueous two-phase systems. *Journal of Fermentation and Bioengineering*.

[16] Willauer H. D., Huddleston J. G., Rogers R. D. (2002). Solute partitioning in aqueous biphasic systems composed of polyethylene glycol and salt: The partitioning of small neutral organic species. *Industrial and Engineering Chemistry Research*.

[17] Anderson J. L., Ding J., Welton T., Armstrong D. W. (2002). Characterizing ionic liquids on the basis of multiple solvation interactions. *Journal of the American Chemical Society*.

[18] Gutowski K. E., Broker G. A., Willauer H. D., Huddleston J. G., Swatloski R. P., Holbrey J. D., Rogers R. D. (2003). Controlling the aqueous miscibility of ionic liquids: aqueous biphasic systems of water-miscible ionic liquids and water-structuring salts for recycle, metathesis, and separations. *Journal of the American Chemical Society*.

[19] Wang Y., Han J., Liu J., Hu Y., Sheng C., Wu Y. (2013). Liquid-liquid equilibrium phase behavior of iminazolium-based ionic liquid aqueous two-phase systems composed of 1-alkyl-3-methyl imidazolium tetrafluoroborate and different electrolytes ZnSO_4_, MgSO_4_ and Li_2_SO_4_ at 298.15 K: experimental and correlation. *Thermochimica Acta*.

[20] Li Y., Zhang M., Su H., Liu Q., Guan W. (2013). Liquid-liquid equilibria of aqueous two-phase systems of the ionic liquid brominated N-ethyl pyridine and sodium dihydrogen phosphate, sodium sulfate, ammonium citrate, and potassium tartrate at different temperatures: Experimental determination and correlation. *Fluid Phase Equilibria*.

[21] Huddleston J. G., Visser A. E., Reichert W. M., Willauer H. D., Broker G. A., Rogers R. D. (2001). Characterization and comparison of hydrophilic and hydrophobic room temperature ionic liquids incorporating the imidazolium cation. *Green Chemistry*.

[22] Albertsson P. A. (1986). Inorganic compound. *Partition of Cell Particles and Macromoleciles*.

[23] Vollhardt D., Fainerman V. B. (2010). Characterisation of phase transition in adsorbed monolayers at the air/water interface. *Advances in Colloid and Interface Science*.

[24] Deanm J. A. (1985). *Lange's Handbook of Chemistry*.

[25] Wuhan University (1995). *Analytical Chemistry. Section 3. Inorganic Compound, 3.12*.

